# Systematic Review on International Salt Reduction Policy in Restaurants

**DOI:** 10.3390/ijerph17249570

**Published:** 2020-12-21

**Authors:** Jingmin Ding, Yuewen Sun, Yuan Li, Jing He, Harriet Sinclair, Wenwen Du, Huijun Wang, Puhong Zhang

**Affiliations:** 1 The George Institute for Global Health at Peking University Health Science Centre, Beijing 100600, China; jding@georgeinstitute.org.cn (J.D.); ysun2@georgeinstitute.org.cn (Y.S.); jhe@georgeinstitute.org.cn (J.H.); zpuhong@georgeinstitute.org.cn (P.Z.); 2 Faculty of Medicine, University of New South Wales, Sydney, NSW 2052, Australia; 3 School of International Studies, American University, Washington, DC 20016, USA; sinclairharrieta@gmail.com; 4 Chinese Center for Disease Control and Prevention, National Institute for Nutrition and Health, Beijing 100050, China; duww@ninh.chinacdc.cn (W.D.); wanghj@ninh.chinacdc.cn (H.W.)

**Keywords:** salt reduction, sodium, restaurant, policy, menu labelling, government initiative, behavior change

## Abstract

As the catering sector has increasingly contributed to population-level salt intake, many countries have begun developing salt-reduction strategies for restaurants. This paper aims to provide an overview of global salt reduction policies in restaurants. Scientific papers and website materials were systematically searched from Web of Science, Science Direct, and PubMed, as well as official websites of government departments and organizations. A total of 78 full-text papers and grey literature works were included. From 58 countries and regions, 62 independent policies were identified, 27 of which were mandatory (3 with fines). The most common strategy was menu labeling, which was a component of 40 policies. Target setting (*n* = 23) and reformulation (*n* = 13) of dishes were also widely implemented. Other salt-reduction strategies included education campaign, chef training, toolkits delivery, table salt removal, media campaign, and government assistance such as free nutrition analysis and toolkits distribution. Most policies focused on chain restaurants. Evaluations of these policies were limited and showed inconsistent results, and more time is needed to demonstrate the clear long-term effects. Attention has been paid to salt reduction in restaurants around the world but is still at its early stage. The feasibility and effectiveness of the strategies need to be further explored.

## 1. Introduction

Excess salt intake has been proved to be a risk factor for non-communicable diseases (NCDs) such as hypertension and cardiovascular disease worldwide [1]. Many countries have developed strategies to reduce population salt consumption since it is one of the most effective measures to reduce the burden of NCDs [2,3,4]. The World Health Organization set a goal of a 30% relative reduction in population intake of salt by 2025 and recommended that daily salt intake should be less than 5 g [5]. The European Union and other countries have followed to make efforts to achieve the salt-reduction goal. China has also taken action to make a 20% reduction in per capita daily salt intake by 2030 [6].

Dietary salt comes from different foods. In developed countries, it was estimated that processed foods contributed most to salt intake [7]. Thus, the salt-reduction strategies in these countries started from the food manufacturing industry and have made significant progress to date. For example, the United Kingdom has set gradual salt reduction targets in processed food from 2006 [8]. In the United States, the government established short-term (2-year) and long-term (10-year) voluntary targets of mean and upper bound sodium levels in processed and prepared food [9].

Meals eaten outside the home make up a large portion of food consumption both in high-income countries and in low- and middle-income countries. In the USA, food purchased from eating out-of-home accounted for more than half of households’ food expenditures in 2018 [10]. In the UK, consumer spending on catering accounted for 28% of food, drink, and catering expenditure in 2017, which has increased by 34% during the past decade [11]. A similar trend has also been observed in Canada, where 83% of young people buy food away from home at least once a week [12]. In China, the Nutrition and Health Monitoring of Chinese Residents in 2010–2012 showed that 42.2% of urban residents had eaten out in the past week before the survey [13]. In India, 38% of adults stated they ate fast food at quick-service restaurants one to three times per week in 2018 [14]. The contribution of restaurant foods to dietary salt intake has become non-trivial. A survey reported that the restaurant foods had the highest sodium density (mg/1000 kcal) among American diets [15]. Therefore, salt reduction in restaurants should be an integral part of overall salt reduction policies.

Several countries have implemented restaurant salt reduction policies. However, many governments have not yet taken action, particularly in developing countries, which have heavier burdens of NCDs compared to high-income countries [16]. Exploring existing restaurant salt reduction experiences will help many countries design and improve relevant strategies. Currently, there are several review papers discussing salt reduction policies [17,18,19], but mainly focusing on the general population and food manufacturing business due to the policy priorities on pre-packaged food. There is thus a gap in the literature regarding salt reduction policies focused on the food service sector. The specific characteristics of the catering industry [20] need to be considered in drawing up policies for restaurants. This study aims to review the restaurant salt reduction policies administered at the national or regional level around the world in order to provide suggestions for effective salt reduction in the catering and restaurant sectors.

## 2. Methods

### 2.1. Search Strategy

Scientific papers referring to relevant policies were searched from Web of Science, Science Direct, and PubMed using keywords “restaurant”, “salt”, “sodium”, or “menu label(l)ing”. The reference lists of included articles were also reviewed for additional sources. Grey literature works were searched from Google and Bing, as well as official websites of government departments (e.g., Ministry of Public Health, Centers for Disease Control and Prevention, Food and Drug Administration) or regional or international organizations (e.g., World Health Organization, European Union, Consensus Action on Salt and Health [21], World Action on Salt and Health [22]), using the same keywords as the literature search. A snowball search strategy was used to find additional relevant materials by accessing links within the website pages.

### 2.2. Inclusion and Exclusion Criteria

Policies, programs, and initiatives issued by the government at all levels (of country and region) regarding reducing salt consumption from restaurants were included in this review. The original policy documents published on the official sites and relevant news reports were included as grey literature. The policies were only considered if they had gone into effect or been passed for implementation. Materials only proposing or negotiating salt reduction without specific strategies were excluded. All materials had to be available in full text in English or Chinese. For policies with multiple available versions, only the latest version was included in this review.

### 2.3. Data Extraction

Key information of each independent policy was compiled into an Excel spreadsheet. The extracted information included the region in which the policy was implemented, enforcement department, date of passage and/or implementation, whether the policy was voluntary or mandatory, main types of strategies, and what the effect was, if any. For the most commonly used strategies, more detailed information, including specific requirements and conditions of restaurants involved, was recorded. Two reviewers conducted data extraction independently and then discussed to reach a consensus. In addition, the PRISMA Checklist was used as a guideline [23].

## 3. Results

**Figure 1:**
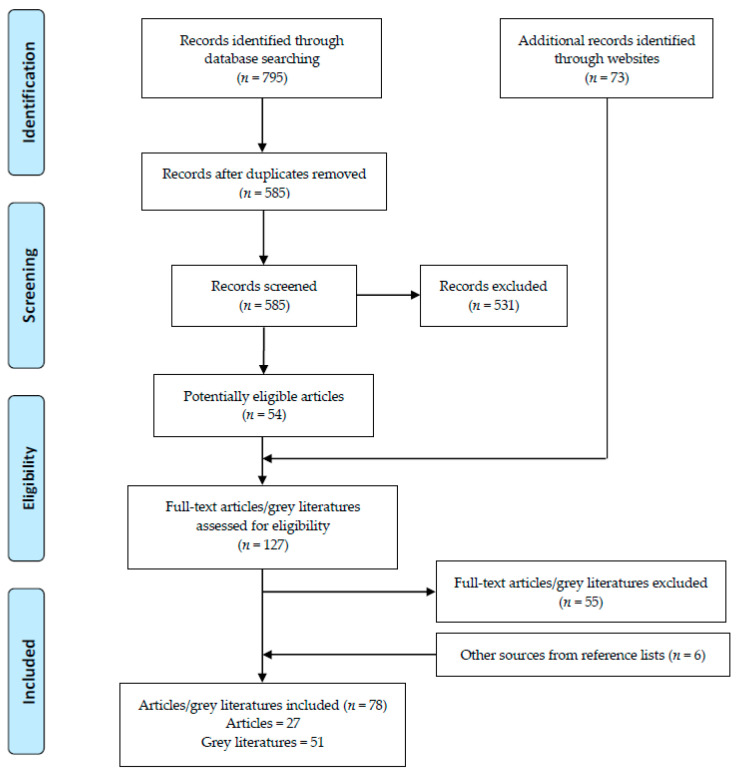
Flow diagram of material selection.

A total of 795 literature papers were detected from scientific databases, of which 585 were screened for eligibility, and then 54 were selected for full-text review. In addition, 73 relevant grey documents were found through a website search. After removing irrelevant materials, 78 papers and grey literature works were included in the analysis (Figure 1). Out of 58 countries and regions, 62 independent policies were identified [9,20,24,25,26,27,28,29,30,31,32,33,34,35,36,37,38,39,40,41,42,43,44,45,46,47,48,49,50,51,52,53,54,55,56,57,58,59,60,61,62,63,64,65,66,67,68,69,70,71,72,73,74,75,76,77,78,79,80,81,82,83,84,85,86,87,88,89,90,91,92,93,94,95,96,97,98,99,100]. Most of these policies were implemented in America and Europe, and about half of the policies detected came from different states or cities within the USA. Strategies were identified and summarized as follows: menu labeling (*n* = 40), target setting (*n* = 23), reformulation of recipes (*n* = 13), consumer education (*n* = 6), chef training (*n* = 4), government assistance (*n* = 3), toolkits delivery (*n* = 2), table salt removal (*n* = 5), and media campaign (*n* = 3) (Table 1). Most policies were introduced and implemented after 2006, and mandatory regulations accounted for about half of these policies (*n* = 27, 3 with fines). Types of restaurants to which the policy covered were mostly chain restaurants with 10 or more outlets.

### 3.1. Menu Labeling

For salt reduction in restaurants, labeling nutrients including sodium of menu items was the most commonly used strategy. Different policies have various criteria for contents labeled on the menu. Besides sodium, calories are the most common nutritional information required for menu labeling, with the aim of obesity prevention [55]. Most relevant policies covered all standard menu items in chain restaurants. Menu labels can take several forms. Six policies mentioned using icons including obvious graphic salt warnings to intuitively display whether the dish is good or bad for health [20,47,48,50,51,52,53,58,65]. For example, the “Keyhole” symbol in Sweden [101] means the food contains less sugar and salt, more fiber and whole grains, or less fat than food products without the symbol in the same product group. In Hong Kong, the “EatSmart Restaurant Star+” Campaign used a colorful mark and stars to indicate the healthy feature of dishes and restaurants. Dish with less fat, salt, and sugar can obtain a “3 Less” mark. Restaurants can get up to three stars by offering “More Fruit and Vegetables” dishes, “3 Less” dishes, and the “EatSmart Promotion” on a daily basis [53].

### 3.2. Target Setting and Reformulation

Some policies set mandatory or recommended salt reduction targets or salt limits for restaurants, and this strategy was often combined with the reformulation of recipes. These strategies were implemented in 32 countries and regions (Table 2). Among them, Australia, Belgium, China, UK, USA, the EU, and the Americas explicitly suggested restaurants to reformulate dishes to meet salt reduction targets. Only six target-setting policies were mandatory, and one in California involved fines. Types of restaurants involved were more diverse, such as quick-service restaurants, cafeterias, and local restaurants. There were 13 policies that set specific salt reduction objectives, which can be divided into two general patterns: limit of maximum sodium content allowed in dishes or meals and goals for salt reduction by a certain percentage over a specific period of time (implemented in Belgium, UK, USA, and the EU). Different regions have different requirements for the upper sodium limit, which varied from 750 mg to 2300 mg sodium per meal. The UK and USA have established category-specific targets for salt levels in restaurant foods [76,84]. For restaurants that sold dishes exceeding the limit, there would be several restrictions, for example prohibiting the sale, requiring warning marks, and restricting giving away incentive items (such as free toys for children’s meals). San Francisco and Shasta County of California State also have special requirements for children’s meals [45,85].

Relevant methods were given to reduce salt in dishes, including reformulating recipes, providing salt separately to the consumer instead of adding salt directly to the meals [75], adjusting salt content of dishes according to the requirements of customers [99], using less sauce or soy sauce, enhancing flavor using additional herbs and spices, and replacing canned vegetables with fresh ones [30]. It is important to reduce salt content in ingredients, since chain restaurants tend to use packaged food (such as cheese and processed meat) to prepare dishes. This requires cooperation with food manufacturers [28,39,40,63,99]. Eight policies have combined restaurant salt reduction with food producers, in terms of involving the whole food industry in the salt reduction process.

### 3.3. Other Strategies

As education is important to build a healthy environment, six policies suggested conducting education campaigns to improve consumer awareness, for example, the harm of high salt diets and instructions on nutritional labels. In addition, China, Thailand, and Philadelphia, and Shasta County in the USA have introduced salt reduction education for restaurant staff and low-sodium cooking training for chefs to improve their knowledge, awareness, and practical skills for salt reduction. Chef training may include: education regarding the effect of salt on health and disease, recommended salt intake, developing several kinds of low salt dishes, practical methods of salt reduction in cooking, and expert demonstration [30,35,91,99].

A total of five policies in Buenos Aires in Argentina, Costa Rica, France, Mexico City in Mexico, and Uruguay recommended that restaurants should provide salt only upon customers’ request, instead of putting salt shakers on the tables [63,73,77]. There were three policies emphasizing the importance of coordination and cooperation with media campaigns, through TV, newspapers, the internet, and roadside advertisement [30,47,51,52,73]. Public health departments can also provide assistance to restaurants in order to effectively promote salt reduction, such as free information sessions and professional consulting [54], free advertising and nutritional analysis for healthy restaurants [35,94], and toolkits distribution (e.g., low-sodium recipe, kitchen utensil, and standard measuring spoon) [30,54]. In the state of New York, local governments created a group purchasing organization to enhance buying power for involved restaurants by increasing purchase volume and decreasing the cost of low-sodium ingredients [35,95].

### 3.4. Effectiveness of Policies

There was limited evidence about the effects of policies after implementation. The results of the existing evaluation were also inconsistent. Of the 17 studies reporting the evaluation after policy implementation, 6 detected positive impacts, 8 showed little or no effect, and 3 described the overall salt reduction effects (not focused on restaurants). The positive impacts mainly came from menu labeling policies, which included the decrease in sodium content in restaurant meals [24,29,30,49,54], the improvement of the salt reduction awareness of chefs and owners [30], and affecting consumer’s choices when purchasing meals with labels [27]. Although it has not yet shown remarkable impact, the menu nutrition information increased the consciousness of the chefs and restaurant operators, as they sometimes changed portion sizes or ingredients after seeing the results of menu analyses [31], or chefs reduced salt when creating new menu items [55,56].

## 4. Discussion

This review provides the first global overview of restaurant salt reduction policies. With the increase in foods consumed out of households, many countries have included the food service sector into their national salt reduction program and gained valuable experience. Population salt reduction requires coordinated efforts from multiple sectors, including restaurants, the food manufacturing industry, and other relevant businesses. Salt and salt-based condiments, with a mainly sensory role, are added to foods to enhance the flavor. Thus, individual intervention may cause a noticeable difference in taste and make salt reduction hard to accept. Therefore, multiple strategies for all foods in the market are more effective to help consumers to form low-salt-eating habits whether at home or out of households by adapting taste buds and enhancing health awareness.

There are many different characteristics between restaurants and food processing industries. The interaction between sellers and consumers is the most important factor, directly affecting the operation of restaurants and policy implementation. Thus, various salt reduction strategies were adopted in different areas, as a one-size-fits-all policy could never fit the needs of all restaurants. The policy orientations can be divided into two types. One is to help consumers make more informed decisions when choosing restaurant foods, which is practiced as information disclosure and education. The other is encouraging restaurants to offer healthier food. This can be achieved by establishing mandatory or voluntary targets and conducting recipe reformulation. The applicability and effectiveness of both buyer- and seller-driven policies will be discussed in the following sections.

### 4.1. Buyer-Driven Policies

Food labels can help consumers identify healthier foods by clearly showing nutrition content or health degree and have been widely utilized in pre-packaged foods [102]. Finland required salt labeling and inclusion of a warning mark for products with excess salt content early in the 1980s [103]. This strategy has just begun to gain popularity among restaurant foods. For example, Sweden expanded the “Keyhole” symbol certification from pre-packaged foods to restaurant meals in 2009 [48]. In the early stage, menu labels included only the amount of calories, aiming to support obesity prevention [104,105], and have now been extended to salt. The current display of the menu label is to list the nutritional information or place a warning icon next to menu items. Colored symbols like “Traffic light” icons and “Nutri-score”, which have been well applied in pre-packaged food [106,107,108], may also be promoted to restaurant menu items.

However, evidence was mixed and inconclusive regarding the impact of menu labeling. Some studies suggested that providing nutrition information of food service items was associated with informed choices for healthier dishes at restaurants [109], as well as the improvement of the health degree of menu items [24,49,110]. On the contrary, some researchers think that labels may cause consumers to choose dishes with unhealthy tag or warning mark as they are often regarded as delicious foods [27,47]. There is also concern that nutrition labels could potentially cause inequality between different socioeconomic groups, since understanding label content and choosing healthier food requires health literacy [111]. In this case, it is necessary to enhance the health literacy of the general public by media campaigns and education [112], to improve the effectiveness of menu labeling strategy.

Although there are few strategies focusing on education in restaurant policies, it is still a priority to raise consumer’s awareness of healthy diet. Among many national or regional salt reduction policies, education for the general public is an important part [113]. That is, consumer education has been integrated into broader health education, instead of aiming at restaurants independently. With a more in-depth understanding of salt reduction, the general public would consciously choose healthier restaurants and dishes.

### 4.2. Seller-Driven Policies

While encouraging consumers to choose healthier options through menu labeling can encourage health-conscious behavior, restaurants themselves also need to make changes to ensure that there are healthy options available. This is primarily achieved by setting an achievable target in order to gradually reduce salt content in meals. Specific targets vary between policies and were set mainly based on national dietary intake recommendations. For example, restaurants in California were asked to claim a recommended sodium intake of less than 2300 mg/d on menus in accordance with US Department of Agriculture guidelines [114], and the New York City salt warning rule required restaurants to place a Salt Shaker warning icon on any dish with more than 2300 mg sodium.

Most of the policies reviewed in this paper set a general upper limit of salt content for all foods on the restaurant’s menu. While based on the experience of the food industry, targeted salt reduction in specific groups of foods would be more effective [40]. A study showed that the progress of salt reduction in restaurants varied depending on food categories [49]. It was found that the salt content in some dishes was even increased and offset by a large reduction in others. For this reason, a general maximum target may be useful in encouraging restaurants to remove dishes with salt content exceeding daily adequate intake level, but this may not work for many other dishes. At present, the UK and the USA have set specific targets for key food categories [76,84]. The Healthy Food Partnership action in Australia also planned to introduce a specific reformulation plan of Quick Service Restaurants [28]. However, establishing category-specific targets like pre-packaged food is demanding work, which would require a comprehensive survey of existing restaurant foods, evaluating their contribution to population salt intake, and monitoring the progress under the oversight of the administration department. This needs to be supported by the popularization of menu labeling laws.

Although some evidence has demonstrated the effectiveness of setting targets and reformulating recipes to reduce salt in restaurants, there are still obstacles to implementing these plans in all types of restaurants. Reformulation and developing new dishes may be challenging and cost more time and money. Thus, the restaurant operators may not be willing to participate in the voluntary initiative, unless making commitments can generate extra profits [115]. Besides, as the revised targets become more challenging over time, participants are more likely to drop out of the agreement. What may help to improve this situation is the support of the food industry. Directly reducing salt content in the ingredients used for food preparation would bypass the technical limits on reformulating menu items. In Canada, food manufactures are asked to apply guiding benchmark sodium reduction levels to all food products, including the ingredients destined for restaurant and foodservice sectors [116].

### 4.3. Applicability of Policies

A unified official policy may not be flexible enough to fit the specific situation of different types of restaurants. Some strategies may work well for a particular type of restaurant but are not suitable for other restaurants, especially those that lack resources and workforce capacity. Therefore, strategies should be developed in line with local conditions. This is why more policies were targeted at chain restaurants, as they had stronger management and standardized menu items that use standard recipes [29]. For example, Canada recommended that restaurants involved in the voluntary policy should have a high degree of standardization [79]. Some less adopted strategies, which perhaps were limited by objective factors to be widely used as a general method, also provided ideas for salt reduction in restaurants. Take chef training, for example: most fast foods served in quick service restaurants are produced by standardized ingredients, so their salt content cannot be changed by chefs, while in Asian countries like China, India, and Thailand, the amount of salt added to dishes is largely determined by the chef when cooking [30,63,117]. Under this circumstance, menu labeling and standardized low-salt menu are less applicable. Policies adopted for these types of restaurants may involve comprehensive aspects like consumer requirements, health education and chef training.

Current policies have taken diverse catering businesses into account but only set restrictions on sit-down meals. Food delivery services, which have popular in recent years, have rarely been mentioned in policy measures [118]. It can be assumed that if a restaurant reformulates recipes and reduces salt content in dishes, the food it delivers would also have low salt content. But there is still the possibility that restaurant menus and takeaway foods are prepared separately in some restaurants. Besides, it is not clear that menu labels can be seen when people order foods on websites or mobile phones. The construction of a healthy environment in restaurants cannot have an impact on these consumers either. Therefore, intervention for delivery food should be introduced in new policy actions. Especially during the COVID-19 pandemic, food delivery service provides a new opportunity for the catering industry, which might be widely adopted by restaurants in the future.

Moreover, in some low- and middle-income countries, foods sold in informal markets play an important role in people’s diets [119,120]. Their safety and health issues should also cause concern, particularly in more vulnerable communities. Strategies that work for chain restaurants like menu labeling and target setting would not be suitable for small businesses, as there are no standardized processes and recipes. Referring to the experience of food safety management [121], health education can be integrated into food hygiene measures among informal food vendors. Meanwhile, guidance for low-salt cooking can be provided together with safe food handling practices.

### 4.4. Evaluation of Policies

Some policies have obtained the support of the catering industry and demonstrated success in salt content and consumer feedback [27,29,30,31,79]. Take Ireland, for example: major chain restaurants have reported reducing salt in meals, and 250 hotels have committed to not adding salt to children’s food at preparation, cooking, or serving stages [62,64]. However, some policies appeared to have had little or no impact [19,24,26,58,78,100]. Most of the salt content information came from menu labels and restaurant websites, while the accuracy of the labels needs to be verified. Overall, the implications of restaurant salt reduction policies remain unclear. A major reason is that, since the national salt reduction initiatives always cover a broader range including the food industry, the food service sector, environment, and mass media [32], it may not be possible to capture the independent contribution of actions undertaken in restaurants among such comprehensive approaches. Similarly, menu labeling focused on restaurants is also combined with calorie labels. Some studies have found that energy content is reduced after labeling regulations, but sodium level is the least likely to meet the requirements [33]. In addition, many policies have been enacted relatively recently, so more time is needed to demonstrate long-term effects. When political popularity is not well-established and sustainable, restaurants may only make a superficial commitment to participate but do not deliver any real change.

This study found that about half of restaurant policies were delivered as mandatory regulations, while others allowed restaurants to participate voluntarily. Evaluation studies showed that mandatory approaches were more likely to achieve positive outcomes, mainly for menu labeling laws [27,29]. However, there are still some limitations. Legislation and supervision take up a considerable amount of national resources and require more time to enact and implement. For policies that require restaurants to make changes, such as reformulating recipes to meet salt reduction targets, it would be more challenging for small businesses. Therefore, target-setting is preferred to be introduced as a voluntary strategy, which provides operators more flexible choices. Although only big enterprises like chain restaurants may be willing and capable to join in the voluntary initiatives, their large market share can achieve a powerful impact and advance the industry towards a healthier direction. In general, a combination of voluntary and mandatory approaches would be better at coping with different intervention measures and regional situations.

### 4.5. Strength and Limitations

This review presents the first international overview of restaurant salt reduction policies using a systematic method. It was found that many developed countries actively promoted salt reduction to the food service sector, and effective strategies have been implemented in several areas internationally. There were few records of developing countries, which may indicate that the salt reduction process is still at an early stage in these countries. However, the scope of inclusion is a limitation. Although we tried to implement a comprehensive strategy, it is possible that some relevant policies might have been missed due to the following reasons. Firstly, we only reviewed English and Chinese publications. As a result, some official policy documents that are not in English/Chinese, as well as studies done by non-English/Chinese speaking researchers, were excluded from this review. Additionally, unlike a database, it is not possible to find exhaustive relevant materials by searching on webpages. There were also link failure issues. In consideration of this problem, we did targeted searches on the national government websites as a supplement to the website searching process.

## 5. Conclusions

Restaurant involvement is an integral part of developing a healthy environment. Along with salt reduction programs in processed food, a series of restaurant salt reduction measures have been developed and have matured in some regions. Two policy orientations were summarized in this review, including buyer-driven and seller-driven policies. Current experiences could be used to explore ideas for policy development and improvement to support uptake in other countries. Further studies on the outcomes of implemented policies would be useful to demonstrate the effectiveness and sustainability of different strategies and then inform future efforts to build a healthier restaurant environment.

## Figures and Tables

**Table 1:** Characteristics of restaurant salt reduction policies implemented in different countries.

Country	Policy	Scope of Implementation	Effective Year	Strategies Adopted	Applicability	Restaurant Type and Size	Effect
Argentina	Less Salt, More Life [63]	Nationwide	2011	Menu labeling, target setting	Mandatory	Unspecified	Unknown
Argentina	An agreement removes salt shakers [77]	Buenos Aires	2011	Table salt removal	Mandatory	Hotel and restaurant	Unknown
Australia	Healthy Food Partnership (a successor to “Food and Health Dialogue”) [28]	Nationwide	2015	Target setting, reformulation, consumer education	Voluntary	Quick service restaurants	Unknown
Belgium	Unspecific [63]	Nationwide	2009	Target setting, reformulation	Voluntary	Unspecified	Unknown
Belgium	EU Salt Reduction Framework * [62,64]	Involved in the EU Framework	2010	Target setting, reformulation	Voluntary	Unspecified	Unknown
Bulgaria	EU Salt Reduction Framework * [62,64]	Involved in the EU Framework	2010	Target setting, reformulation	Voluntary	Unspecified	Unknown
Canada	Health Canada’s Healthy Eating Strategy [25]	Nationwide	2003	Menu labeling	Voluntary	Restaurants and food services establishments	A study compared laboratory values with Nutrition Facts table (NFt) values of foods from supermarkets, bakeries, and restaurants showed: 16.7% (*n* = 169) of foods exceeding ±20% of the NFt Sodium was the least accurate content (*n* = 49, 18.4%), all underreported than laboratory values
Canada	Sodium Reduction Strategy for Canada [49,79]	Nationwide	2010	Menu labeling, target setting	Voluntary	Restaurants and food services establishments with a high degree of standardization	−25 mg (*p* < 0.001) sodium per serving overall; There were increases and decreases in different kinds of food; −220 mg (a decline of 19%) in reduced-sodium foods; No change in percentage of foods exceeding recommended sodium intake (1500 mg and 2300 mg per day)
Canada	Informed Dining program (IDP) [24,59,60,61]	Province of British Columbia	2012	Menu labeling	Voluntary	Range from small independent cafes to national restaurant chains	Little or no impact
China	Healthy Restaurant (in National Healthy Lifestyle Action) [99]	Nationwide	2013	Menu labeling, reformulation, consumer education, chef training	Voluntary	Unspecified	Unknown
China	“EatSmart Restaurant Star+” Campaign [53]	Hong Kong	Unknown	Menu labeling	Voluntary	Unspecified	Unknown
Costa Rica	Unspecific [73]	Nationwide	Unknown	Table salt removal	Voluntary	Unspecified	Unknown
Finland	Reducing salt intake in populations ** [54]	Nationwide	2006	Menu labeling, consumer education, government assistance, toolkits delivery	Voluntary	Caterers, restaurants and others involved in commercial meal preparation	Unknown
Finland	EU Salt Reduction Framework * [62,64]	Involved in the EU Framework	2010	Target setting, reformulation	Voluntary	Unspecified	Unknown
Finland	Unspecific [63,64]	Nationwide	2011	Target setting	Mandatory	University restaurants (main meals and all meal components)	Unknown
France	Reducing salt intake in populations ** [54]	Nationwide	2006	Menu labeling, consumer education, government assistance, toolkits delivery	Voluntary	Caterers, restaurants and others involved in commercial meal preparation	Unknown
France	Unspecific [63]	Nationwide	Unspecific	Table salt removal	Voluntary	Unspecified	Unknown
Greece	EU Salt Reduction Framework * [62,64]	Involved in the EU Framework	2010	Target setting, reformulation	Voluntary	Unspecified	Unknown
Hungary	EU Salt Reduction Framework * [62,64]	Involved in the EU Framework	2010	Target setting, reformulation	Voluntary	Unspecified	Unknown
Ireland	Reducing salt intake in populations ** [54]	Nationwide	2006	Menu labeling, consumer education, government assistance, toolkits delivery	Voluntary	Caterers, restaurants, and others involved in commercial meal preparation	Major high-street restaurant chains all reported a greater reduction in salt from products.
Latvia	EU Salt Reduction Framework * [62,64]	Involved in the EU Framework	2010	Target setting, reformulation	Voluntary	Unspecified	Unknown
Lithuania	EU Salt Reduction Framework * [62,64]	Involved in the EU Framework	2010	Target setting, reformulation	Voluntary	Unspecified	Unknown
Mexico	− Sal + Salud [73]	Mexico City	Unknown	Table salt removal, media campaign	Voluntary	Unspecified	Unknown
Netherlands	EU Salt Reduction Framework * [62,64]	Involved in the EU Framework	2010	Target setting, reformulation	Voluntary	Unspecified	Unknown
Portugal	EU Salt Reduction Framework * [62,64]	Involved in the EU Framework	2010	Target setting, reformulation	Voluntary	Unspecified	Unknown
Romania	EU Salt Reduction Framework * [62,64]	Involved in the EU Framework	2010	Target setting, reformulation	Voluntary	Unspecified	Unknown
Slovak Republic	EU Salt Reduction Framework * [62,64]	Involved in the EU Framework	2010	Target setting, reformulation	Voluntary	Unspecified	Unknown
Slovenia	EU Salt Reduction Framework * [62,64]	Involved in the EU Framework	2010	Target setting, reformulation	Voluntary	Unspecified	Unknown
Spain	Reducing salt intake in populations ** [54]	Nationwide	2006	Menu labeling, consumer education, government assistance, toolkits delivery	Voluntary	Caterers, restaurants, and others involved in commercial meal preparation	Unknown
Sri Lanka	Unspecific [75]	Nationwide	Unknown	Reformulation	Unspecified	Food outlets and restaurants	Unknown
Sweden	Keyhole label [48]	Nationwide	2009	Menu labeling	Voluntary	Unspecified	The Keyhole symbol is widely recognized by Swedish consumers and has been promoted to restaurants
Thailand	Thai Food, Good Heart [63]	Nationwide	2004	Reformulation, chef training	Voluntary	Thai Food restaurants (10 famous Thai dishes)	Unknown
UK	UK Food Standards Agency’s (FSA) salt reduction programme [39,40,98]	Nationwide	2003	Target setting, reformulation	Voluntary	Catering sector, quick-service restaurants, workplace caterers, pub and high-street restaurant chains, and coffee and sandwich shops	Overall salt reduction effects: population’s average daily salt intake was reduced from 9.5 g in 2000–2001 to 8.6 g in 2008. Some foods reduced salt levels by up to 70%.
UK	Reducing salt intake in populations ** [54]	Nationwide	2006	Menu labeling, consumer education, government assistance, toolkits delivery	Voluntary	Caterers, restaurants, and others involved in commercial meal preparation	Unknown
UK	New Department of Health (DoH) voluntary targets [74]	Nationwide	2014	Target setting	Voluntary	Unspecified	Unknown
UK	The Public Health Responsibility Deal (RD) in England [36,44,76]	England	2017	Target setting, reformulation	Voluntary	Unspecified	Unknown
UK	EU Salt Reduction Framework * [62,64]	Wales (involved in the EU Framework)	2010	Target setting, reformulation	Voluntary	Unspecified	Unknown
Uruguay	Unspecific [63]	Nationwide	Unknown	Menu labeling, reformulation, table salt removal	Mandatory	Unspecified	Unknown
USA	Strategies to Reduce Sodium Intake in the United States [100]	Nationwide	2008	Menu labeling, target setting	Mandatory	Large, multiunit chain restaurant/foodservice operations	Unknown
USA	Menu Labeling Provisions (of Section 4205 of the Patient Protection and Affordable Care Act) [19,20,26]	Nationwide	2010	Menu labeling	Mandatory	Chain restaurants and similar retail food establishments with 20 or more locations	No significant change in mean sodium overall; −70 mg mean sodium across all restaurants in added vs. removed menu items at the 75th percentile
USA	Statement of Policy Salt Reduction [81]	Nationwide	2014	Menu labeling, target setting	Voluntary	Unspecified	Unknown
USA	Nutrition Labeling of Standard Menu Items in Restaurants and Similar Retail Food Establishments [55,56]	Nationwide	2015	Menu labeling	Mandatory	Chain restaurant with 20 or more locations offering for sale substantially the same menu items	Mean sodium did not change significantly; New menu items reduced 70 mg sodium on average than old ones
USA	Unspecific [78]	Nationwide	2015	Reformulation	Voluntary	Unspecified	A study assessing children’s menus reported that the sodium target was the least frequently met healthy criteria
USA	Voluntary Sodium Reduction Goals [70]	Nationwide	2016	Target setting	Voluntary	Unspecified	Unknown
USA	Heart Check [35]	Nationwide	Unknown	Menu labeling, target setting,	Voluntary	Unspecified	Unknown
USA	New US healthcare reform law [44]	Nationwide	Unknown	Menu labeling	Mandatory	Retail food establishments with 20 locations	Unknown
USA	National Salt Reduction Initiative (NSRI) [20,32,33,83,84]	New York with 100 city and state health authorities and national health organizations	2010	Target setting, reformulation	Voluntary	Unspecified	Minor effect on sodium levels in top chain restaurant foods; Sodium far exceeded recommended limits
USA	SB-1420 Food facilities: nutritional information [50]	California	2009	Menu labeling, target setting	Mandatory (fines imposed)	Chain restaurants with 20 or more outlets in California	Unknown
USA	Smart Meal [35,86]	Colorado	Unknown	Menu labeling, target setting	Voluntary	Local restaurants	Unknown
USA	Strategies to Reduce Sodium Intake in the United States (bill) [100]	Delaware	2010	Menu labeling, consumer education	Mandatory	Foodservice establishment with 10 or more outlets in Delaware or nationwide	Unknown
USA	Strategies to Reduce Sodium Intake in the United States [100]	District of Columbia	2010	Menu labeling	Mandatory	Chain restaurants with 10 or more outlets nationwide	Unknown
USA	Healthy Choices [35,93]	Erie County	Unknown	Menu labeling	Voluntary	Local restaurants	Unknown
USA	Strategies to Reduce Sodium Intake in the United States [100]	Florida	2010	Menu labeling	Mandatory	Chain restaurants with 19 or more outlets in Florida	Unknown
USA	Strategies to Reduce Sodium Intake in the United States [100]	Indiana	2009	Menu labeling	Mandatory	Chain restaurants of 20 or more outlets in Indiana	Unknown
USA	Strategies to Reduce Sodium Intake in the United States [100]	Kentucky	2009	Menu labeling	Mandatory	Chain restaurants with 10 or more locations in Kentucky	Unknown
USA	The King County Board of Health’s nutrition labeling regulation [29,43,69]	King County	2010	Menu labeling	Mandatory (fines imposed, was voluntary between 2008–2010)	Chain restaurant meets all criteria: has 15 or more locations in King County or nationwide; has operating permits; total gross annual revenues of $1 million or more; has standardized menu items that use standard recipes	−231 ± 727 mg (*p* < 0.01) in sodium for all entrees at sit-down chains (*n* = 11); Levels still above the recommended limits (one-third of the nutrient intake recommendations)
USA	Strategies to Reduce Sodium Intake in the United States [100]	Maryland	2010	Menu labeling	Mandatory	Chain restaurants with 15 or more outlets nationwide	Unknown
USA	Healthy Howard Columbia [94]	Maryland	Unknown	Government assistance	Voluntary	Local restaurants	Unknown
USA	The menu labeling requirement [100]	Montgomery County	2010	Menu labeling	Mandatory	Chain restaurants with 20 or more outlets nationwide	Unknown
USA	New York City salt warning rule [47,51,52]	New York	2015	Menu labeling, target setting, media campaign, consumer education	Mandatory	Chain restaurant with 15 outlets	The effect of adding numeric sodium menu nutrition information depends on consumers’ taste intuition
USA	Capitol Region Restaurant Operators Cooperative [35,95]	New York	Unknown	Government assistance	Voluntary	Local restaurants	Unknown
USA	Winner’s Circle [35,88]	North Carolina	Unknown	Menu labeling, target setting	Unspecified	Local restaurants	Unknown
USA	Strategies to Reduce Sodium Intake in the United States [100]	Oklahoma	2010	Menu labeling	Mandatory	Restaurants with 10 or more outlets in the state	Unknown
USA	State legislation [100]	Oregon	2010	Menu labeling	Mandatory	Chain restaurants with 15 or more outlets nationwide	Unknown
USA	Strategies to Reduce Sodium Intake in the United States [100]	Pennsylvania	2009	Menu labeling	Mandatory	Chain restaurants with an average of at least $500,000 in food sales over the past 3 years	Unknown
USA	Philadelphia Menu Labeling Ordinance [27]	Philadelphia	2010	Menu labeling	Mandatory	Restaurants with 15 or more outlets nationwide	Consumers at labeled restaurants purchased 224 mg less sodium than at unlabeled restaurants; Consumers who reported menu label affecting their choices purchased 370 mg less sodium
USA	Healthy Chinese Take-Out Initiative (HCTI) [30]	Philadelphia	2012	Reformulation, chef training, toolkits delivery, media campaign	Voluntary	Chinese take-out restaurants in low-income urban communities	13–34% reduction in sodium content of 3 popular dishes 36 months after low-sodium cooking training
USA	Sodium menu labeling legislation [65]	Philadelphia	2019	Menu labeling	Mandatory (fines imposed)	Any chain retail food establishment	Unknown
USA	SmartMenu [31,34]	Pierce County	2007	Menu labeling	Voluntary	Locally owned full-service restaurants	−45 mg sodium in the average entree after labeling; About 1/3 patrons reported behavior change because of seeing nutrition information
USA	¡Por Vida! [35,87]	San Antonio	Unknown	Menu labeling, target setting	Unspecified	Local restaurants	Unknown
USA	Healthy Food Incentives Ordinance [37,45,96]	San Francisco	2011	Target setting (for children’s meals)	Mandatory	Local restaurants	Two restaurants investigated did not change recipes to meet the nutrition criteria (only selling toys separately from children’s meals as response to the ordinance)
USA	Sodium Savvy [35,90]	Schenectady County	Unknown	Menu labeling, target setting, consumer education	Unspecified	Local restaurants	Unknown
USA	Healthy Kids Choice [35,85]	Shasta County	Unknown	Menu labeling, target setting	Unspecified	Local restaurants	Unknown
USA	Cut the Sodium but Keep the Flavor [35,91]	Shasta County	Unknown	Chef training	Voluntary	Local restaurants	Unknown
USA	Strategies to Reduce Sodium Intake in the United States [100]	Tennessee	2009	Menu labeling	Mandatory	Chain restaurants with 20 or more outlets nationwide	Unknown
USA	Strategies to Reduce Sodium Intake in the United States [100]	Texas	2010	Menu labeling	Mandatory	Chain restaurants with 19 or more locations in Texas	Unknown
USA	Strategies to Reduce Sodium Intake in the United States [100]	Vermont	2010	Menu labeling	Mandatory	Restaurants with 10 or more outlets nationwide	Unknown
Americas ^1^	Preventing Cardiovascular Disease in the Americas by Reducing Dietary Salt Intake Population-Wide [72]	Countries in Americas	2014	Target setting, reformulation	Voluntary	Unspecified	Unknown

* Count as one policy. ** Count as one policy. ^1^ One exception of heading: Americas is not a country.

**Table 2:** Policy-driven targets in different countries and regions.

Country and Region	Target/Limit	Food Manufacturer Involved *
Americas	Gradual and sustained schedules	Yes
Argentina	Unspecified	No
Australia	Unspecified	Yes
Belgium	10% salt reduction by 2012	No
Canada	Unspecified (specific target only for the food industry)	Yes
China	Unspecified	Yes
EU (Belgium, Bulgaria, Finland, Greece, Hungary, Latvia, Lithuania, Netherlands, Portugal, Romania, Slovak Republic, Slovenia, and UK: Wales)	16% salt reduction over 4 years	Yes
Finland	Unspecified	No
Thailand	Unspecified (for 10 famous Thai dishes)	Yes
UK	Specific target for 11 restaurant food categories (24 subcategories) within 2 years	Yes
USA	Specific target for 10 restaurant food categories (25 subcategories) in 2012 and 2014	Yes
California, USA	Stating 2300 mg of sodium limits for daily diet	No
Colorado, USA	≤1500 mg sodium per meal and ≤650 mg sodium per side dish	No
New York, USA	Menu items containing 2300 mg sodium or more are required to add Salt Shaker icon	No
North Carolina, USA	≤1000 mg sodium per meal and ≤480 mg sodium per side item	No
San Antonio, USA	≤750 mg sodium per meal	No
San Francisco, USA	≤640 mg sodium for children’s meal	No
Schenectady County, USA	≤750 mg sodium per meal; ≤250 mg sodium for appetizers, side dishes, and desserts	No
Shasta County, USA	≤770 mg sodium for children’s meal	No

(*) To reduce restaurant meal salt content by reducing food ingredients produced by food manufacturers.
